# Climatic variables influence the temporal dynamics of an anuran metacommunity in a nonstationary way

**DOI:** 10.1002/ece3.6217

**Published:** 2020-04-03

**Authors:** Karoline Ceron, Diego J. Santana, Elaine M. Lucas, Jairo José Zocche, Diogo B. Provete

**Affiliations:** ^1^ Instituto de Biociências Universidade Federal de Mato Grosso do Sul Campo Grande Brazil; ^2^ Departamento de Zootecnia e Ciências Biológicas Universidade Federal de Santa Maria Palmeira das Missões Brazil; ^3^ Pós‐Graduação em Ciências Ambientais Universidade do Extremo Sul Catarinense Criciúma Brazil; ^4^ Gothenburg Global Biodiversity Centre Göteborg Sweden

**Keywords:** Atlantic Forest, Santa Catarina, Serra Furada State Park, STATICO, storage effect

## Abstract

Understanding the temporal dynamics of communities is crucial to predict how communities respond to climate change. Several factors can promote variation in phenology among species, including tracking of seasonal resources, adaptive responses to other species, demographic stochasticity, and physiological constraints. The activities of ectothermic vertebrates are sensitive to climatic variations due to the effect of temperature and humidity on species physiology. However, most studies on temporal dynamics have analyzed multi‐year data and do not have resolution to discriminate within‐year patterns that can determine community assembly cycles. Here, we tested the temporal stability and synchrony of calling activity and also how climatic variables influence anuran species composition throughout the year in a metacommunity in the Atlantic Forest of southern Brazil. Using a multivariate method, we described how the relationship between species composition and climatic variables changes through time. The metacommunity showed a weak synchronous spatial pattern, meaning that species responded independently to environmental variation. Interestingly, species composition exhibited a nonstationary response to climate, suggesting that climate affects species composition differently depending on the season. The species‐climate relationship was stronger during the spring, summer, and winter, mainly influenced by temperature, rainfall, and humidity. Thus, temporal community dynamics seem to be mediated by species life‐history traits, in which independent fluctuations promote community stability in temporally varying environments.

## Graphical abstract

Using a multivariate method called STATICO, we described the stable patterns and the temporal changes of the relationship between species composition and the climatic variables. The metacommunity showed a weak synchronous spatial pattern, meaning that species responded independently to environmental variation. Interestingly, species composition exhibited a nonstationary response to climate, suggesting that climate affects species composition differently depending on the season.

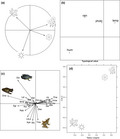

## 1 INTRODUCTION

Time in ecology has been usually considered as a niche axis (Post, 2019) along with space and food (Schoener, 1974; Toft, 1985), all of which are prone to be partitioned by organisms. Time can also be viewed as a resource (Post, 2019). However, differently from space and food, the use of time by an individual does not make it unavailable to other individuals. Instead, the partitioning of time occurs within an individual, since the allocation of time to a given life‐history phase (or phenophase) makes it unavailable to another phenophase (Post, 2019). For organisms whose life span comprehends more than one annual cycle, the decision of when to exhibit a given life‐history phase is critical to determine its fitness (Cayuela et al., 2014; Forrest & Miller‐Rushing, 2010; Post, 2019). Reproduction is a life‐history event tightly linked to fitness. As such, natural selection favors individuals that are better at predicting the future environment and decide if it is time to invest energy on reproduction (Visser et al., 2010).

The phenology of a species involves the temporal coupling of vital activities (e.g., reproduction, migration) to environmental conditions (Canavero, Arim, Pérez, Jaksic, & Marquet, 2019; Visser et al., 2010). The choices of allocation of time to a given life‐history event may vary among individuals and species (Post, 2019). In fact, studies commonly report that species in the same community respond to different environmental cues (e.g., Kopp & Eterovick, 2006; Steen et al., 2013). This pattern can be explained by the distinct limits of tolerance of co‐occurring species, which evolved in response to abiotic or biotic selective agents (Post, 2019). Environmental cues that indicate the appearance of favorable environmental conditions or resources upon which organisms rely for reproduction, dispersal, and foraging are potential abiotic selective agents determining phenological patterns. The most important environmental cues are climatic variables, such as temperature, rainfall or humidity (Post, 2019), and nonclimatic factors, such as light and prey availability (Canavero et al., 2019). The presence or intensity of these variables trigger physiological responses at the individual level, which in turn influence the timing of life‐history events (Chmura et al., 2019; Visser et al., 2010).

Day length (or photoperiod) is used as a cue to indicate the time of the year, which can point to the future availability of resources (Canavero & Arim, 2009; Forrest & Miller‐Rushing, 2010). However, photoperiod alone may not provide a feasible cue for appropriate weather conditions. As a result, organisms usually respond to multiple climatic variables, such as temperature and rainfall (Post, 2019). Both temperature and photoperiod are relevant environmental cues in temperate environments (Canavero & Arim, 2009; Canavero, Arim, & Brazeiro, 2009; While & Uller, 2014), whereas rainfall or humidity becomes more important drivers of phenological patterns in tropical and arid areas (Cohen, Lajeunesse, & Rohr, 2018; Schalk & Saenz, 2016). Also, aquatic organisms usually use rainfall as a predictive cue to the presence and duration of water on ephemeral habitats, like ponds (Jared et al., 2019).

Another approach to investigate temporal community dynamics is to measure how their stability and synchrony changes through time. Stability and synchrony through time have been usually investigated at the population level, but recent approaches have proposed metrics to quantity them across ecological hierarchies (see Wang et al., 2019). A stable system is able to withstand small but frequent perturbations (Schreiber, 2006). Population synchrony patterns reflect the response of species abundance to environmental factors (Wang et al., 2019). Synchronicity happens when populations respond to environmental factors in the same way. As a result, populations that exhibit high synchrony are more susceptible to local extinction due to extreme environmental phenomena, such as frosts. Understanding how population synchrony varies throughout space is key to understand metapopulation stability and how species persist in the landscape (Liebhold et al., 2004). Accordingly, highly unstable populations of co‐occurring species can promote stability at the community level. In particular, asynchrony among species can result in community stability if a decline in one species is compensated by an increase in abundance in another (Loreau & De Mazancourt, 2008). Dispersal is a key process that stabilizes local communities, while also contributing to increase their synchrony throughout space (Liebhold et al., 2004). Therefore, local communities that are more strongly connected by species dispersal are expected to exhibit higher synchrony. Conversely, asynchrony can arise from dispersal limitation generating compensatory dynamics at the community level, that is, when groups of species increase in abundance while others decrease. However, few studies have explored how spatial synchrony influences population maintenance in terrestrial, dispersal‐limited organisms (e.g., Desharnais, Reuman, Costantino, & Cohen, 2018). Environmental heterogeneity can also buffer populations of ectotherms against climatic variation, since heterogeneous habitats have a wider range of microhabitats with diverse microclimates and resources (McCaffery et al., 2014; Oliver et al., 2010; Piha et al., 2007). Thus, population dynamics can potentially impact species interaction and drive patterns at the community scale.

Amphibians seem to be the animals most affected by climate change (Post, 2019; While & Uller, 2014), with the highest average shift toward earlier breeding in several locations (Todd et al., 2010). At the same time, amphibians are partially flexible in terms of decisions of time allocation (Todd et al., 2010). For example, their breeding period may vary from one year to the next in response to the seasonality in abiotic variables (Cayuela et al., 2014; Vaira, 2005) or as a response to density‐dependent interactions, resulting in priority effects promoted by the sequence of arrival time of species to a given habitat (Rudolf, 2019). Hence, amphibians are an ideal group to investigate the effects of environmental change on patterns at the population and community level (see Todd et al., 2010). However, most papers dealing with phenological patterns in breeding activity of amphibians have only analyzed aggregated community metrics, by relating species abundance and richness to climatic variables (reviewed in Wells, 2007). This approach can mask how different species respond to variations in climate through time. Conversely, species composition data allow us to examine how phenological strategies vary within a community, as a consequence of species life‐history traits.

While questions about how the relationship between species distribution and environmental parameters changes through time has been explored in water bugs (Slimani et al., 2017), mayflies (Thioulouse, 2011), streams invertebrates (Thioulouse et al., 2018), and fish (Kidé et al., 2015), the factors that affect the temporal dynamics of communities of terrestrial ectothermic vertebrates in terms of their species composition are less known. This is essential to understand key ecological patterns, like the importance of abiotic factors to community structure.

Here, we analyzed the breeding phenology of a frog metacommunity throughout four seasons in temperate South America asking the following questions: (a) How do climatic variables influence species composition in communities? We expect species to vary in their sensitivity to climatic variables due to their life‐history traits, resulting in distinct temporal dynamics among species, that is, asynchrony. (b) Are species abundances stable through time and space, that is, do communities show a perfect synchrony among species? We expect high calling activity fluctuation throughout the year, due to the strong seasonality of the study area, resulting in nonstable communities, because anuran calling activity is strongly influenced by climatic variables, like temperature and humidity.

## 2 MATERIAL AND METHODS

### 2.1 Study area

We conducted fieldwork at the Serra Furada State Park in Orleans and Grão‐Pará, Santa Catarina, southern Brazil (28°11′07.77″S, 49°23′31.80″W; DATUM = WGS84; Figure S1). Elevation ranges from 360 to 1,000 m a.s.l., and the vegetation consists of remnants of submontane, montane, and high montane tropical rain forests of different degrees of conservation, ranging from initial to advanced stages of succession (FATMA, 2010). The climate is temperate humid with hot summers (Alvares et al., 2013) with average temperatures between 17.0°C and 19.3°C and minimum and maximum average temperatures ranging from 12.0°C to 25.9°C. It can occur from 0.3 to 11.0 frosts per year. The relative humidity varies from 81.4% to 82.2%. Annual precipitation ranges from 1,200 to 1,700 mm, with rainfall well distributed throughout the year. Seasons are composed by a hot and wet summer (December–March), a dry and cold winter (June–September), a relatively dry autumn (March–June), and a relatively hot spring (September–December) (EPAGRI, 2001).

### 2.2 Sampling design

We conducted monthly surveys for two consecutive days in six sites in two areas from August 2014 to July 2015. Elevation in the south area varies from 360 to 700 m. This area has remnants of submontane and montane tropical rain forests. Elevation in the north area varies from 800 to 1,000 m. This area has remnants of montane tropical rain forests. For the analysis, we grouped data from April through June as autumn, October to December as spring, January through March as summer, and July through September as winter. The sampling days were chosen randomly each month. We established six 100‐m transects: two transects in forest streams, two in forest trails, and two in marshes in open areas, which were located both in south and north areas (one transect in each environment per area). These environments were chosen in order to maximize the breath of habitats used by anurans found in the sampling area. On each sampling day, we surveyed the set of environments (one stream, forest, and marsh) in each area (south or north). We sampled frogs three times a day in each environment for 2 hr using survey at breeding sites (Crump & Scott, 1994) and acoustic survey techniques (Zimmerman, 1994), between 1,500 hr and 0000 hr for a total of 144 hr of sampling.

We counted the number of calling males in each site to estimate seasonal calling activity, following Gottsberger and Gruber (2004). We did not use mark‐recapture sampling design. Thus, we are aware that our calling activity estimates do not account for repeated measures, which might bias the detection of temporal patterns (Banks‐Leite, Ewers, Pimentel, & Metzger, 2012). However, we did not model species abundance per se, but the number of calling males of each species in each site through time.

### 2.3 Climatic variables

We obtained climate data (temperature, accumulated rainfall, and relative humidity) from a weather station 55 km apart from the study site. The distance between the weather station and our study site can be problematic for climate data. However, this is only true for regions with high topographical heterogeneity (see Graae et al., 2012), which is not the case, since the study site and the site in which the weather station is located share the same vegetation type and mountain chain. Temperature, rainfall, and humidity data consisted of 3‐day averages per month (2 days before and the day of sampling). We obtained photoperiod data for each day (in minutes of daylight) from the Brazilian National Observatory (http://staff.on.br/jlkm/ephemeris/index.php). We checked for correlation between climatic variables using VIF in package *usdm* (Naimi, 2017) in R v. 3.1.3 (R Core Team, 2017).

### 2.4 Data analysis

#### 2.4.1 Community stability through time

To test for community stability, we calculated the rate of community change and synchrony following Gross et al. (2014) for each site using the R package *codyn* (Hallett et al., 2016). The metric is given by the formula: $\eta =\left(1/n\right)\sum_{i}corr\left(Y_{i^{\prime }}\sum_{j\neq i}Y_{j}\right)$ , where *Y_i_* is the biomass of species *i* in a group of *n* species, which is the average across species of the correlation between the biomass of each species and the total biomass of all other species in the group. Gross' *η* is a synchrony metric standardized from −1 (perfect asynchrony) to 1 (perfect synchrony) and is centered at 0 when species fluctuate independently in the community (Gross et al., 2014). The rate of community change reflects the continual reshuffling of species within the same community (temporal species turnover).

#### 2.4.2 Influence of climatic variables on community structure through time

To analyze how the species‐environment relationship changes through time, we used STATICO (STATIS and CO‐inertia; Thioulouse et al., 2004). This method performs a multivariate ordination with four sets of paired matrices (Thioulouse, 2011) to describe the stable patterns and the spatio‐temporal changes of the relationships between species composition and climatic variables. Our data consist of a set of two paired matrices for each season: one site by species and the other site by climatic variables.

**FIGURE 1:**
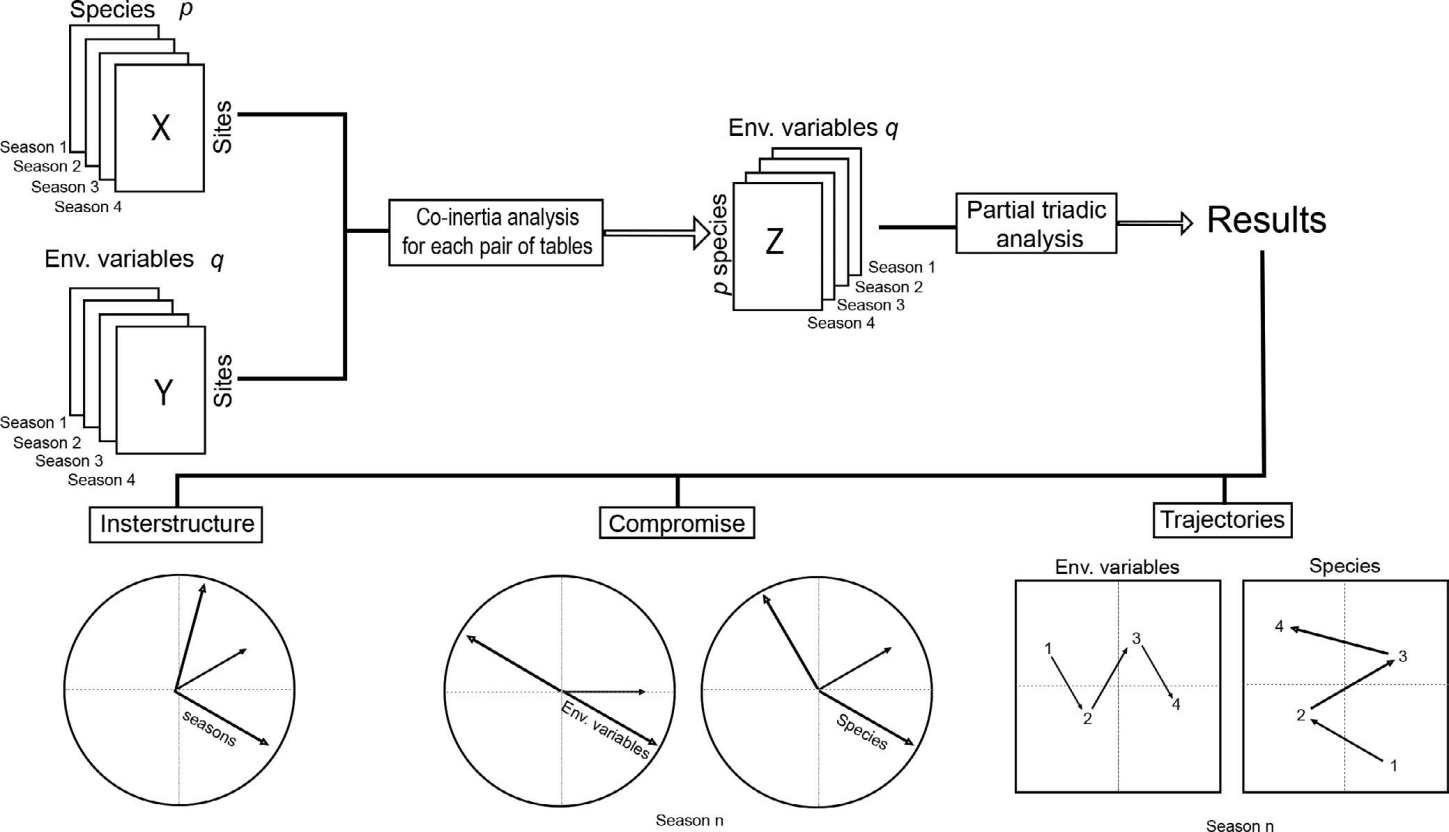
Flowchart showing the STATICO analysis modified from Kidé et al. (2015). The data are a sequence of *n* pairs of ecological tables. The *X* and *Y* are respectively the pairs of tables for species composition and climatic variables. *Z* is the cross‐table with *p* number of species and *q* number of climatic variables. The analysis proceeds by first conducting a weighted principal component analysis (PCA) on the climatic variables matrix and a centered correspondence analysis (CA) on the species composition matrix. Then, a Co‐inertia analysis is used to link the pairs of PCA‐CA, producing a sequence of cross‐tables. Finally, a Partial Triadic Analysis (PTA) is used to analyze this series of cross‐tables

STATICO proceeds in three stages: (a) The first stage consists in analyzing each matrix by a one‐table method (weighted principal components analysis of the environmental variables and centered correspondence analysis of the species data, in our case); (b) each pair of tables is then analyzed by a Co‐inertia analysis (Dolédec & Chessel, 1994; Dray, Chessel, & Thioulouse, 2003), which is a two‐matrix coupling method, allowing a cross‐matrix to be computed between the variables of the two matrices (here between species composition and climatic variables); (c) finally, a Partial Triadic Analysis (Thioulouse & Chessel, 1987) is used to analyze this paired sequence of matrices (Figure 1). STATICO generates three plots (Figure 1): the interstructure, the compromise, and the intrastructure analyses. The interstructure identifies the similarity between each pair of matrices and displays for each season the similarity among environmental variables, species, and sites. The compromise is an ordination plot of the environmental variables and species in a common reduced space. This step shows the stable part of the average species‐environment relationships across seasons. Variation in the length of arrows in different seasons indicates variation in species‐environment relationship across seasons. The trajectory maps project species and environmental variables for each season as additional elements on the compromise axes, in order to summarize the reproducibility of the structure across the series of cross‐matrices (see Kidé et al., 2015). Analysis was conducted in the *ade4* package (Dray & Dufour, 2007) in R v. 3.1.3 (R Core Team, 2017). Raw data, along with R code used to run the analysis, are deposited at FigShare (Ceron, Santana, Gonsales, Zocche, & Provete, 2019).

## 3 RESULTS

We recorded 25 anuran species during our study period (Figure S2). The calling activity peaked from October to January. *Boana bischoffi* had the longest calling period, calling for 9 months consecutively. *Aplastodiscus ehrhardti*, *B. bischoffi*, and *Vitreorana uranoscopa* were the only species that called during the coldest months (May to July). There were more species of calling males in the summer (*n* = 24), than spring (*n* = 21), winter (*n* = 14), and autumn (*n* = 13). Spring had more calling individuals (*n* = 325) than summer (*n* = 215), winter (*n* = 76), and autumn (*n* = 63).

**FIGURE 2:**
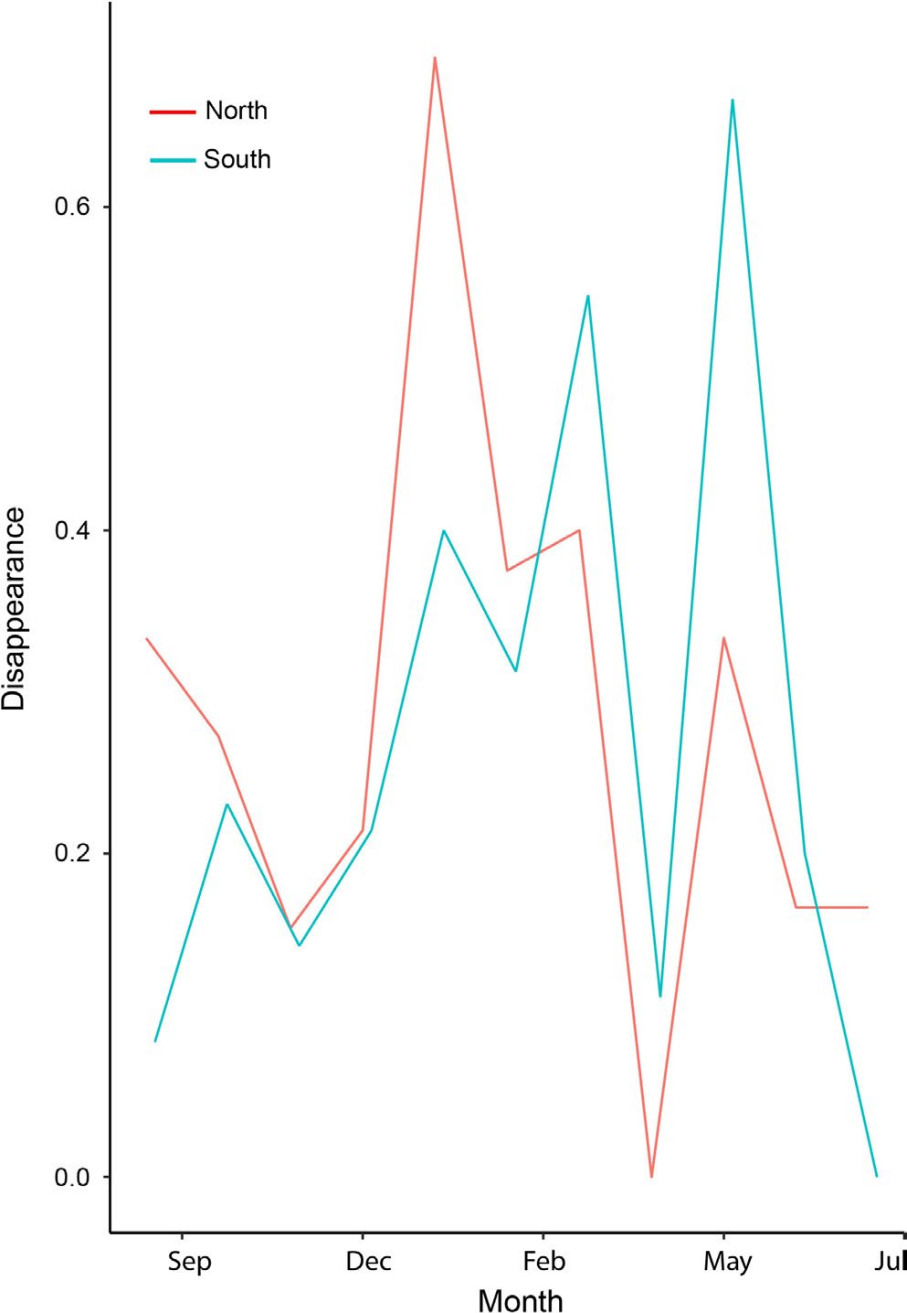
Species turnover throughout the year as a proportion of lost species (disappearance) relative to the total species observed across the time period for the south and north areas

Species composition varied both spatially and temporally. All climatic variables exhibited a clear seasonal pattern throughout the sampling period (Figure S3). Species synchrony in most sites was close to zero in both areas (north = 0.12, south = 0.3), suggesting that the number of calling males is fluctuating independently when we considered the whole sampling period. The areas showed an asynchronous pattern of species turnover, with the north area showing a peak of species disappearance in January, while the species disappearance in the south area peaks in May (Figure 2). Accordingly, the north area had higher values of stability (1.94) than the south one (1.12).

**FIGURE 3:**
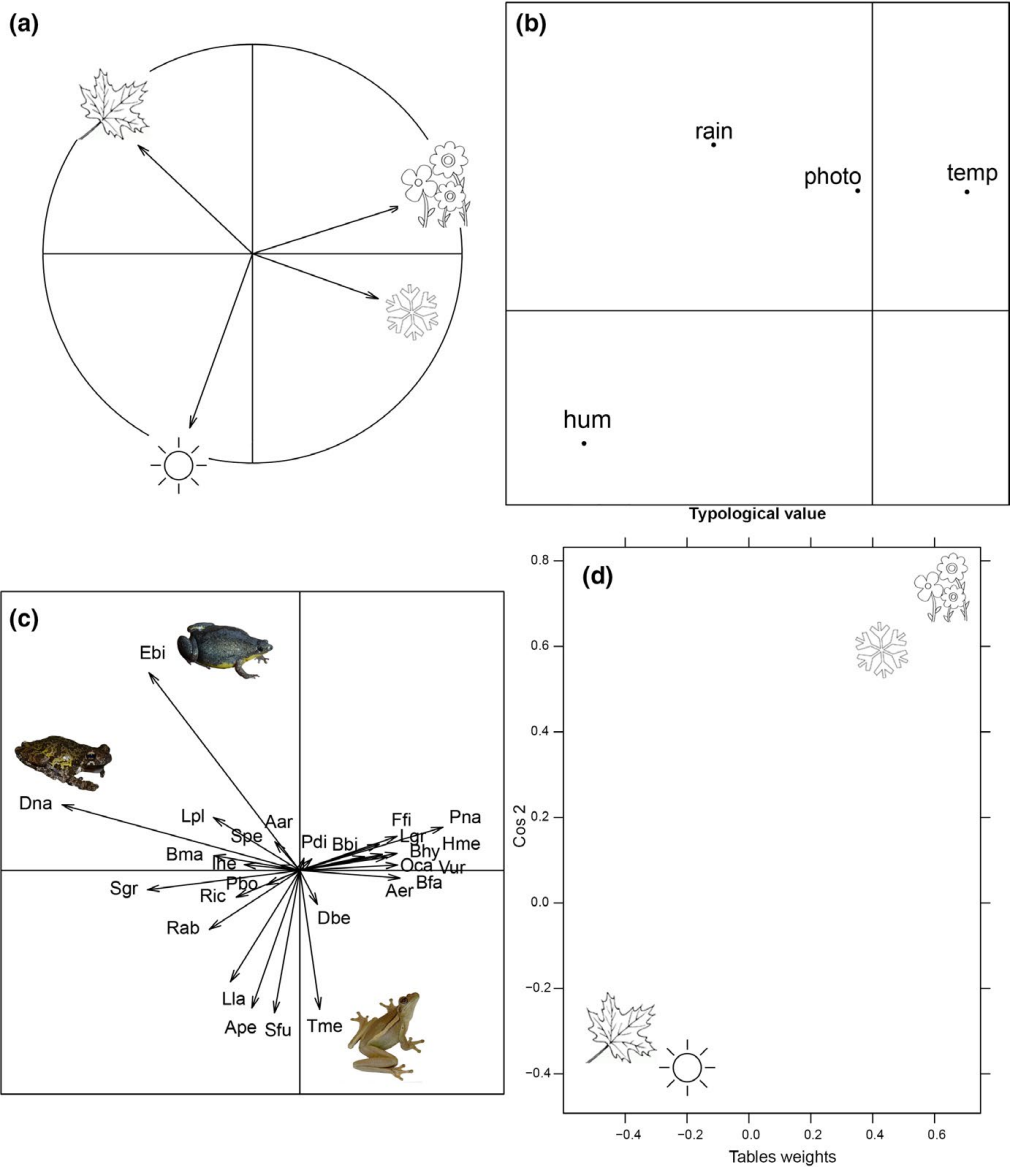
Interstructure and compromise factor maps of the STATICO analysis. These ordination diagrams show anurans–climatic variables relationships: (a) Importance of each season to the relationship between climatic parameters and species preferences; (b) climatic variables projected on the first factorial plan, where: Photo = photoperiod, Rain = rainfall, Hum = humidity, temp = temperature; (c) Hellinger‐transformed abundances of anuran species projected on the same factorial plan (See Table S1 for species name abbreviations); (d) typological value of seasons and their influence in climatic parameters and species preferences (square cosines vs table weights)

Winter and spring were more similar to each other in terms of species composition (Figure 3a) and had the highest influence in the compromise, the diagram that represents species‐environment relationships, because these seasons had higher weights in species response to environment (Figure 3d). The first axis was mostly related to temperature, while the second axis was related to rain (Figure 3b). Conversely, the summer and autumn were negatively related to the first axis and the second axis had the lowest influence in the compromise (Figure 3b,d). Similarity in species composition between seasons was mainly driven by species response to temperature, rain, and photoperiod (Figure 3b), although individual species showed different responses to climatic variables (Figure 3c). For example, the breeding activity of *Elachistocleis bicolor* was positively influenced by rain, while *Trachycephalus mesophaeus* was influenced negatively by photoperiod, but the majority of species were little affected by climatic variables (notice that most arrows are short in Figure 3).

**FIGURE 4:**
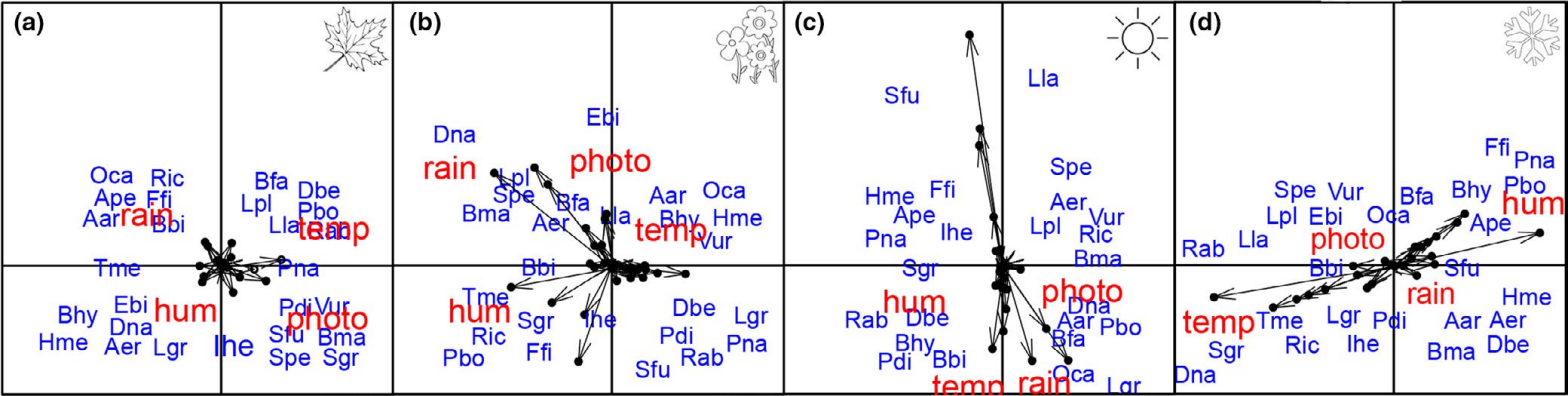
Interstructure plot of STATICO for climatic variables (red) and species (blue). Photo = photoperiod, rain = rainfall, hum = humidity, temp = temperature. Arrow length represents the strength of the relationship. Notice that arrows are longer in the winter (d) and spring (b), and summer (c) than in autumn (a), indicating a variation in the strength of the species‐environment relationship throughout the year

**FIGURE 5:**
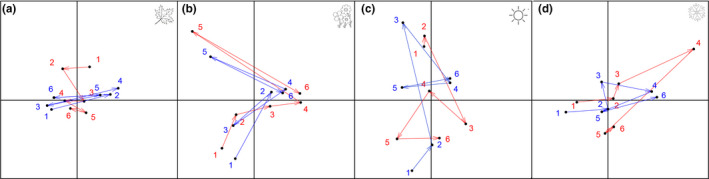
Trajectory plot of STATICO sampling sites for climatic variables (red) and species (blue). Numbers indicate sampling sites grouped by seasons: Autumn (a), summer (b), spring (c), and winter (d) (see Material and Methods). Notice that the direction of arrows in the pairs of figures point to different directions in different seasons, suggesting that species respond differently to climate depending on the season

Species composition was weakly correlated with climatic variables during the autumn (Figure 4a). However, in the summer, spring, and winter species composition was influenced mostly by temperature, rainfall, and rainfall + humidity, respectively (Figure 4). In the winter, some species were negatively, while others were positively associated with temperature and humidity (Figure 4d). For example, the calling activity of *Physalaemus nanus* increased with an increase of humidity in the winter and the activity of *Ololygon catharinensis* was related to rain during the summer (Figure 4). However, the relative importance of climatic variables to some species changed with seasons. For example, the calling activity of *Dendropsophus nahdereri* was related to temperature in the winter, but to rainfall in the summer. Similarly, species composition seems not to track climatic variables during autumn and summer, because trajectories of sites in the ordination diagram of climatic variables do not mirror the trajectory in the species composition diagram (Figure 5). During these seasons, community similarity did not reflect the rate of change in climatic variables. Conversely, species seem to track climatic variables more strongly during the winter and spring, changing positions in the ordination diagram following changes in climatic variables (Figure 5). Taken together, these results show a nonstationary pattern in the species‐environment relationship throughout the year.

## 4 DISCUSSION

Our results show that species are fluctuating independently in the metacommunity, and species composition exhibit a nonstationary response to climate, which was mainly related to temperature, rainfall, and humidity during the spring, summer, and winter. Calling activity varied temporally, with the majority of species calling from October to January. These results show that differences in limits of physiological performance are producing diverse patterns of reproductive activity of anurans throughout the year.

Species composition in local communities varied independently throughout the year, showing a weak synchronous pattern. Synchrony results from fluctuations in population densities among sites correlated over time (Desharnais et al., 2018). The lack of synchrony among sites can be caused by the different requirements of species, resulting in distinct responses to environmental conditions (Magurran & Henderson, 2010). The breeding activity of anurans is strongly related to the variance in abiotic conditions, such as temperature and rainfall (Duellman & Trueb, 1986). However, some species in the studied metacommunity had different environmental requirements mainly due to their reproductive modes (see Haddad & Prado, 2005), making species abundance to fluctuate independently. For example, some species are usually less sensitive to variation in humidity and precipitation, such as *Fritziana mitus* that lives in water‐holding bromeliads and *Ischnocnema henselii* that is a direct developer (Haddad & Prado, 2005). Conversely, the majority of species depend on water for reproduction, but they differ in breeding strategy (Wells, 2007) and reproductive modes, with some species calling during long periods (continuous breeders; e.g., *B. bischoffi*) and other species calling for short periods during the wet season (explosive breeders; e.g., *Rhinella icterica*). Thus, environmental conditions vary across space, with different conditions favoring different species promoting an independent fluctuation, as predicted by the storage effect (Chesson, 1986).

Species composition was correlated with temperature, humidity, and rainfall during the spring and summer, whereas some species were affected differently by temperature and humidity during the winter. The thermal tolerance of species creates a gradient of activity along the year, with cold‐adapted species (e.g., *B. bischoffi* and *V. uranoscopa*; Gouveia, Dobrovolski, Lemes, Cassemiro, & Diniz‐Filho, 2013) calling in cold months, while other species reduce their performance during colder months (Kiss et al., 2009). This temperature gradient was clear in the winter, when the calling activity of some species was positively related to low temperatures and high humidity (e.g., cold‐adapted species). Temperature limitation in ectotherms can be explained by the oxygen‐limited thermal tolerance hypothesis (Winwood‐Smith et al., 2015). This hypothesis postulates that thermal limits (i.e,. the minimal and maximal temperatures at which an organism can survive) in ectotherms are determined by the inability of the circulatory system to deliver oxygen above and beyond an organism's basal metabolic requirements at low and high temperatures. This difference between maximal oxygen supply and the metabolic cost of basic physiological maintenance (aerobic scope) allows an organism to devote energy to additional functions, such as locomotion and reproduction (Carey, 1979). Temperature is a well‐documented environmental cue that affects anuran calling activity (see review in Duellman & Trueb, 1986). High levels of humidity and rainfall decrease the risk of desiccation during breeding activity, since anurans have highly permeable skin (Blaustein, Wake, & Sousa, 1994). Additionally, high relative humidity may also aid in call transmission, since sound travels more efficiently through humid than dry air (Harris, 1966). In addition, explosive breeders are less responsive to abiotic environment than prolonged breeders, because of the very short breeding periods (Oseen & Wassersug, 2002). For example, *Proceratophrys boiei* and *Trachycephalus mesophaeus* are explosive breeders and called for 3 and 1 months, respectively, whereas *B. bischoffi* and *A. ehrhardti* are prolonged breeders calling for 11 and 7 months, respectively. Additionally, the complex life cycle of anurans may allow populations to persist even in temporally heterogeneous environments, for example, tadpoles that stay longer in ponds than adults and metamorphose in the next season. This can be similar to a “seed bank,” buffering populations against extinction, allowing a storage effect. These differences in life‐history traits and thermal tolerances make species composition related to multiple variables.

We found a seasonal pattern in the climatic variables. Seasonal environments impose fluctuating selection on life‐history traits that can show different responses among species (Varpe, 2017). The phenology of an organism is necessary to maintain each life stage inside the limits of physiological performance in seasonal environments (Varpe, 2017). However, subtropical regions show a weak seasonality (Canavero et al., 2009), because temperature hardly changes, and rainfall is well distributed throughout the year. This weak seasonality allows communities to be more stable than in highly seasonal environments, since cold‐adapted species replace noncold‐adapted species during cold months. The lower stability of the south area in relation to north area was probably due to the altitude difference between them, which also resulted in differences of species disappearance. The north area has a higher elevation (up to 100 m), and it is colder than the south area. The former area contains species adapted to cold temperatures (such as *B. bischoffi* and *Dendropsophus nahdereri*), whereas the latter area has more generalists species but not many cold‐adapted ones. This difference in species composition influenced species disappearance throughout the year, with south and north areas showing different peaks. In addition, adaptations to cold climate of species occurring in the northern area make the community more stable in response to climatic fluctuations.

Species seem to track climatic variables during the summer, spring, and winter, but less so during autumn. The main difference among these seasons was temperature, which was lower during the autumn and higher in the summer and spring. These results support the idea of temperature as the main determinant of community structure in subtropical regions (Canavero et al., 2009). Conversely, our results showing low influence of photoperiod on metacommunity structure differ from other studies in subtropical regions in South America (Both, Kaefer, Santos, & Cechin, 2008; Canavero & Arim, 2009). During the winter and autumn, the availability of both resources and reproductive habitats decreases due to lower temperatures, leading the majority of species to minimize energy use, using estivation or hibernation, where animals experienced long periods without food (Valenzuela‐Sánchez et al., 2015). Only cold‐adapted species are active during this period, since their circulatory system is able to delivery oxygen to the whole organism even in low temperatures (Kiss et al., 2009), allowing locomotion and reproduction. Thus, community patterns seem to be mediated by species life‐history traits, in which independent fluctuations promote community stability in temporally varying environments.

## CONFLICT OF INTEREST

None declared.

## AUTHOR CONTRIBUTIONS

**Karoline Ceron**: Conceptualization (equal); formal analysis (equal); investigation (lead); writing – original draft (lead); writing – review & editing (lead). **Diego J. Santana**: Data curation (equal); writing – original draft (equal); writing – review & editing (equal). **Elaine M. Lucas**: Conceptualization (equal); writing – original draft (equal). **Jairo José Zocche**: Conceptualization (equal); writing – original draft (equal). **Diogo B. Provete**: Conceptualization (lead); formal analysis (lead); methodology (lead); writing – original draft (equal); writing – review & editing (equal).

## Supporting information

Fig S1

Fig S2

Fig S3

Table S1

Fig S1‐S3_captions

## Data Availability

The data used in this study is available at FigShare Digital Repository at https://figshare.com/s/14191f2216c297b9ab69.
