# Supplementary figures and images for: Climatic variables influence the temporal dynamics of an anuran metacommunity in a nonstationary way
Source: Ecol Evol. 2020 Apr 3;10(11):4630–9. doi: 10.1002/ece3.6217 (PMC7297772; doi:10.1002/ece3.6217)

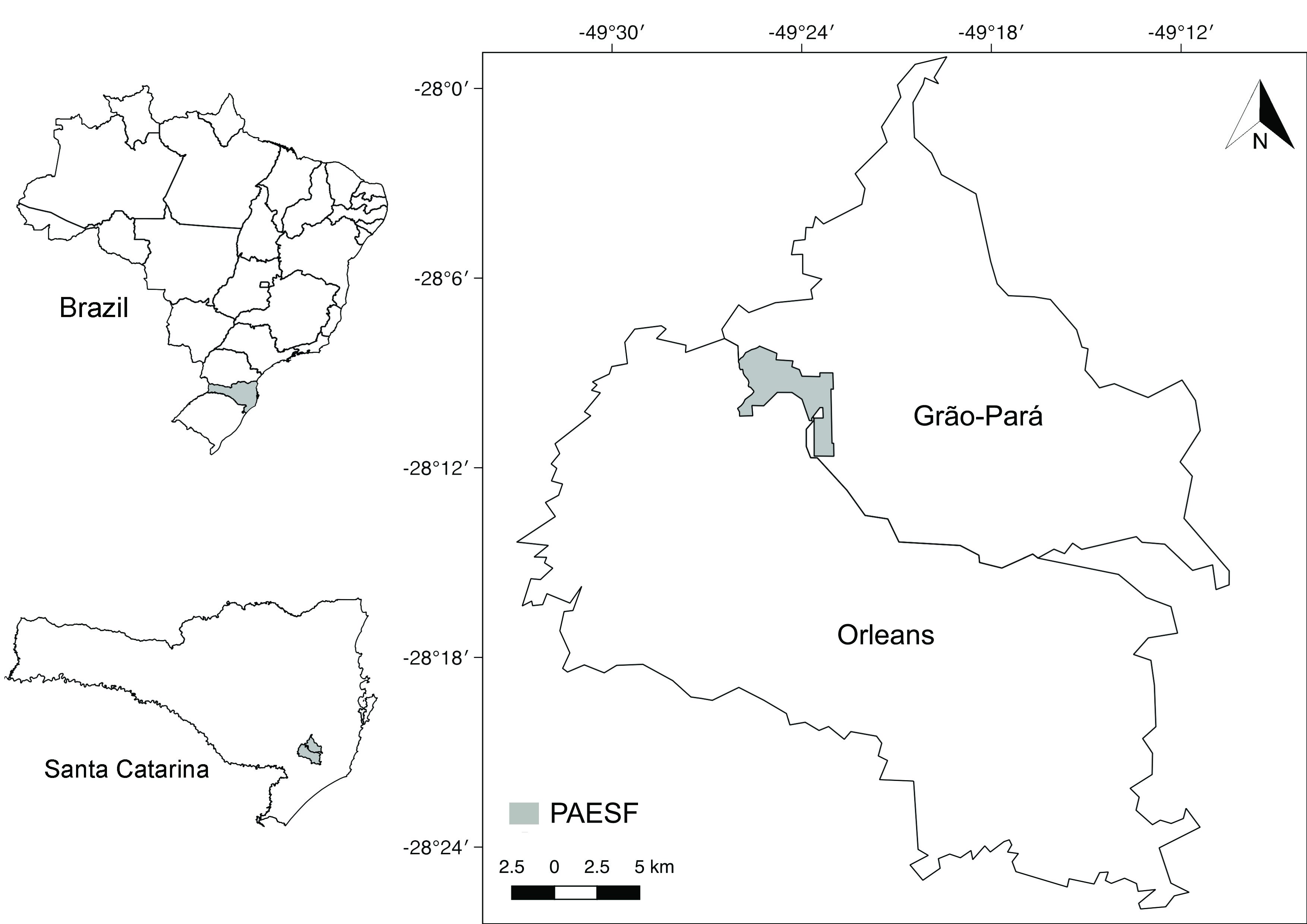

Supplement: Supplementary file 1 — Fig S1 [file ECE3-10-4630-s001.jpg]

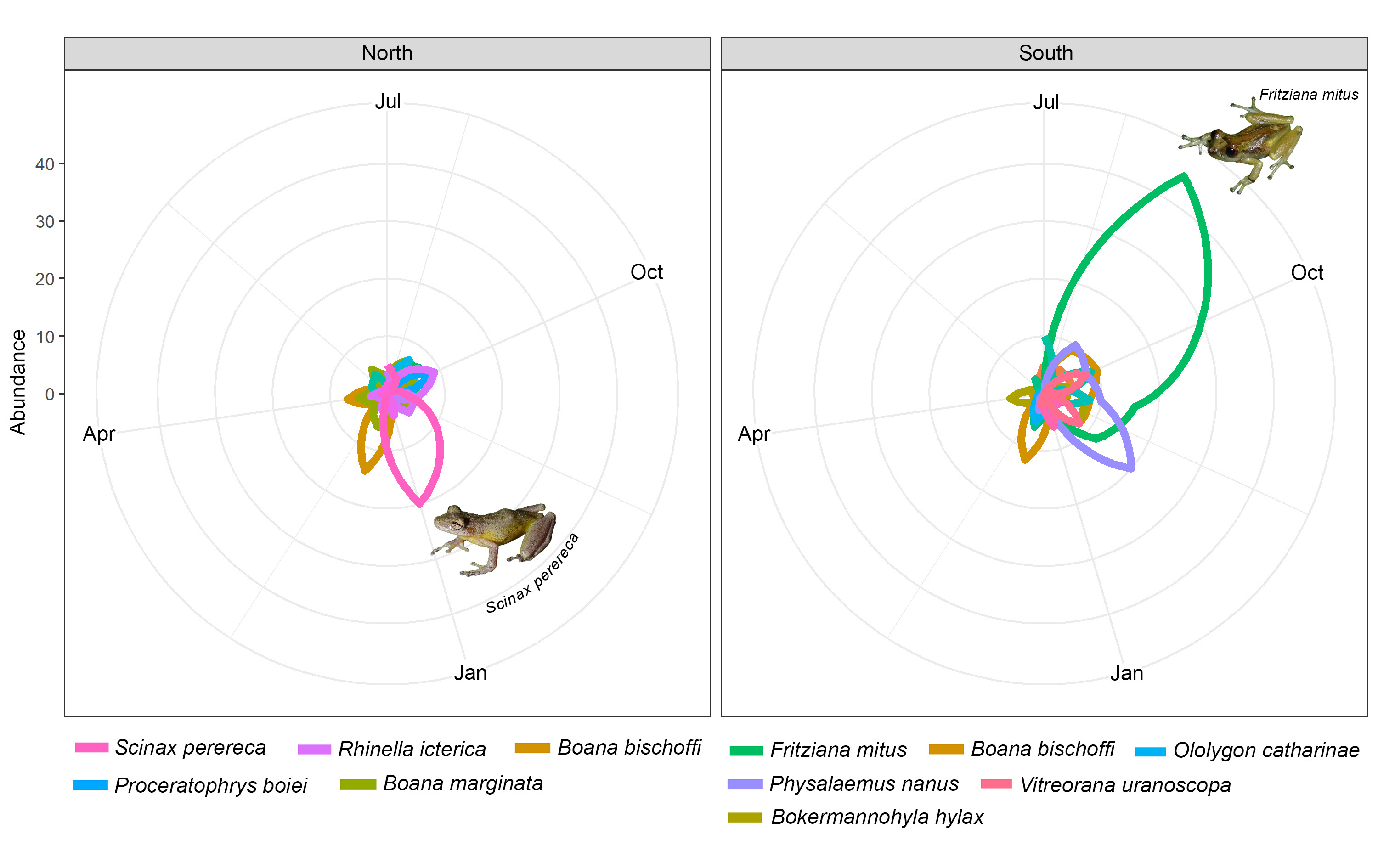

Supplement: Supplementary file 2 — Fig S2 [file ECE3-10-4630-s002.jpg]

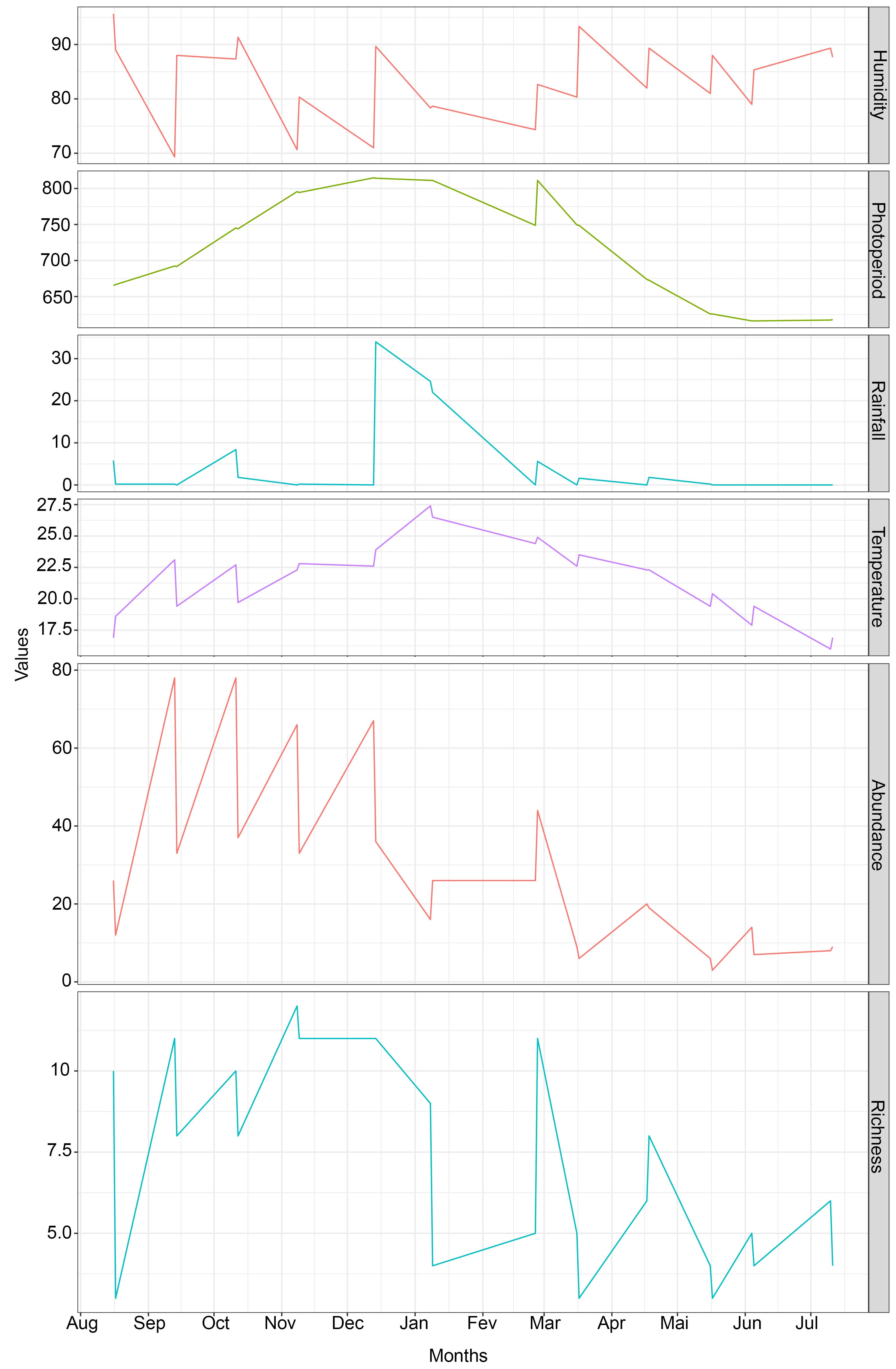

Supplement: Supplementary file 3 — Fig S3 [file ECE3-10-4630-s003.jpg]
