# Supplementary material for: Climatic variables influence the temporal dynamics of an anuran metacommunity in a nonstationary way
Source: Ecol Evol. 2020 Apr 3;10(11):4630–9. doi: 10.1002/ece3.6217 (PMC7297772; doi:10.1002/ece3.6217)
Supplement: Supplementary file 4 — Table S1 [file ECE3-10-4630-s004.docx]

TABLE S1. Anuran species abbreviation.

| Abbreviation | Species |
| --- | --- |
| Aer | *Aplastodiscus ehrhardti* (Müller, 1924) |
| Ape | *Aplastodiscus perviridis* A. Lutz in B. Lutz, 1950 |
| Bhy | *Bokermannohyla hylax* (Heyer, 1985) |
| Dbe | *Dendrophryniscus berthalutzae* Izecksohn, 1994 “1993” |
| Dna | *Dendropsophus nahdereri* (B. Lutz & Bokermann, 1963) |
| Ebi | *Elachistocleis bicolor* (Valenciennes in Guérin-Menéville,1838) |
| Fmi | *Fritziana mitus W*alker, Wachlevski, Nogueira da Costa, Nogueira-Costa, Garcia, and Haddad, 2018 |
| Hme | *Hylodes meridionalis* (Mertens, 1927) |
| Bbi | *Boana bischoffi* (Boulenger, 1887) |
| Bfa | *Boana faber* (Wied-Neuwied, 1821) |
| Bma | *Boana marginata*(Boulenger, 1887) |
| Ihe | *Ischnocnema henselii* (Peters, 1872) |
| Aar | *Adenomera araucaria* Kwet & Angulo, 2003 |
| Lgr | *Leptodactylus gracilis* (Duméril & Bibron, 1841) |
| Lla | *Leptodactylus latrans* (Steffen, 1815) |
| Lpl | *Leptodactylus plaumanni* Ahl, 1936 |
| Pdi | *Phyllomedusa distincta* A. Lutz in B. Lutz, 1950 |
| Pna | *Physalaemus nanus* (Boulenger, 1888) |
| Pbo | *Proceratophrys boiei* (Wied-Neuwied, 1825) |
| Rab | *Rhinella abei* (Baldissera-Jr,Caramaschi & Haddad, 2004) |
| Ric | *Rhinella icterica* (Spix, 1824) |
| Oca | *Ololygon catharinae* (Boulenger, 1888) |
| Sfu | *Scinax fuscovarius* (A. Lutz, 1925) |
| Sgr | *Scinax granulatus* (Peters, 1871) |
| Spe | *Scinax perereca* Pombal, Haddad & Kasahara, 1995 |
| Tme | *Trachycephalus mesophaeus* (Hensel, 1867) |
| Vur | *Vitreorana uranoscopa* (Müller, 1924) |
