# Supplementary material for: Climatic variables influence the temporal dynamics of an anuran metacommunity in a nonstationary way
Source: Ecol Evol. 2020 Apr 3;10(11):4630–9. doi: 10.1002/ece3.6217 (PMC7297772; doi:10.1002/ece3.6217)
Supplement: Supplementary file 5 — Fig S1‐S3_captions [file ECE3-10-4630-s005.docx]

# APPENDIX S1

FIGURE S1**.** Map showing the study site in the state of Santa Catarina, southern Brazil. PAESF stands for Serra Furada State Park.

FIGURE S2. Temporal variation in abundance of anuran calling activity in north and south areas.

FIGURE S3. Variation in climatic variables and species composition throughout the sampling period from August 2014 to July 2015.
